# Interleukin-22 ameliorates liver fibrosis through miR-200a/beta-catenin

**DOI:** 10.1038/srep36436

**Published:** 2016-11-07

**Authors:** Bang-li Hu, Cheng Shi, Rong-e Lei, Dong-hong Lu, Wei Luo, Shan-yu Qin, You Zhou, Hai-xing Jiang

**Affiliations:** 1 Department of Gastroenterology, the First Affiliated Hospital of Guangxi Medical University, Nanning 530021, China; 2 Systems Immunity University Research Institute, Cardiff University School of Medicine, Heath Park, Cardiff, UK

## Abstract

IL-22 ameliorates liver fibrosis by inhibiting hepatic stellate cells (HSC), and loss of miR-200a is associated with the development of liver fibrosis. The study aimed to investigate the interplay between IL-22 and miR-200a in regulating liver fibrosis *in vivo* and *in vitro*. We observed that IL-22 significantly reduced the proliferation of HSC and increased the expression of p-STAT3. β-catenin was identified as a target gene of miR-200a by luciferase reporter assay, and upregulation of miR-200a significantly attenuated the proliferation of HSC and reduced β-catenin expression. IL-22 treatment increased expression of miR-200a and decreased expression of β-catenin in HSC. The expression of p-STAT3 and miR-200a was elevated while β-catenin was decreased in fibrotic rat liver after IL-22 treatment. Expression levels of β-catenin and p-STAT3 were inversely correlated in fibrotic rat liver and HSC. Upregulation of β-catenin suppressed expression of p-STAT3 in HSC. We concluded that IL-22 inhibits HSC activation and ameliorates liver fibrosis through enhancing expression of miR-200a and reducing expression of β-catenin, suggesting there may be a crosstalk between IL-22/STAT3 and β-catenin pathway.

Liver fibrosis, a primary consequence of chronic liver injury, is characterized by activation of hepatic stellate cells (HSC) and accumulation of extracellular matrix and collagen protein. It has been suggested that inhibiting HSC activation is an important strategy for the treatment of liver fibrosis^1^,^2^. The pathogenesis of liver fibrosis is regulated by a variety of inflammatory cytokines. Interleukin-22 (IL-22), a critical immune mediator, has been reported to ameliorate liver fibrogenesis in mice by inducing the senescence of HSCs^3^. Moreover, our study also showed that treatment with IL-22 accelerated the resolution of liver fibrosis in mice^4^.

MicroRNAs (miRNAs) are short, non-coding RNA molecules that regulate gene expression via mRNA deadenylation or destabilization and translational repression or activation. miRNAs regulate a variety of cellular processes including cell proliferation, differentiation, invasion, migration, and epithelial-mesenchymal transition^5^. Increasing evidence shows a few miRNAs act as important regulators in the pathogenesis of liver fibrosis^6^,^7^. It has been recently reported that increasing expression of miRNA-200a attenuates HSC proliferation while knocking down miR-200a prompted HSC proliferation^8^,^9^.

Both IL-22 and miRNA-200a alleviate the pathogenesis of liver fibrosis. However, whether there is any crosstalk between IL-22 and miRNA-200a in anti-fibrotic effects remains unknown. In the present study, we searched potential target of miR-200a and explored the mechanism of IL-22 and miR-200a in inhibiting HSC activity. We further examined the regulatory role of IL-22 in the expression of miR-200a and its target in a HSC cell line, primary HSCs and a rat model of liver fibrosis. Our results reveal the interaction between IL-22 and miR-200a in regulating the development of hepatic fibrosis.

## Material and Methods

### Animals and treatments

The study protocol was approved by the ethics committee of the First Affiliated Hospital of Guangxi Medical University. All experimental procedures on rats were approved by the ethics committee of Animal Experiments of Guangxi Medical University. The study was carried out in accordance with the recommendations in the Guide for the Care and Use of Laboratory Animals of the National Institutes of Health. Normal male Sprague–Dawley rats (100–120 g) were obtained from Laboratory Animal Center (Guangxi Medical University, China, N.O. SCXKG 2010-0002). Animals were kept in the pathogen-free animal room (12 hours light/12 hours dark; temperature, 22–24 °C), and received water ad libitumin in the Animal Care Facility Service (Guangxi Medical University, China).

### Administration with recombinant IL-22 protein

To induce the liver fibrosis, rats were intraperitoneally (IP) injected with 1 mL/kg body weight of 50% CCl4 in olive oil 2 times weekly for 8 weeks. After this, mice (n = 10) were injected IP with 0.3 μg/g body weight of recombinant rat interleukin-22 (rrIL-22) (R&D Systems, Inc., Minneapolis, USA) once per week for 2 weeks. Mice (n = 10) administered with 0.5% BSA in PBS were used as controls.

### Knocking-down miR-200a

To knock down the expression of miR-200a, rats with liver fibrosis (n = 10) were hydrodynamicaly tail vein injected with lent-miR-200a inhibitors (1 × 10^8^ TU/mL) in PBS for 1 week. After this, rats were IP injected with 0.3 μg/g body weight of rrIL-22 once per week for 2 weeks. After the scarification, the liver tissues were collected and fixed in 10% neutral buffered formalin for hematoxylin and eosin (H&E) staining and Masson staining.

### Histology and immunohistochemistry

Slices of tissue were prepared in 4 μm thickness and stained with H&E and Masson staining according to standard procedures. Two experienced pathologists assessed liver histology blindly by using light microscopy (Nikon Eclipse E800 Microscope, Kawasaki, Kanagawa, Japan). For immunohistochemistry, the sections were incubated with primary antibody of α-SMA (1:300 dilution, Sigma-Aldrich), followed by incubation with streptavidin-peroxidase complex. Peroxidase conjugates were subsequently visualized using diaminobenzidine solution. The sections were then counterstained with hematoxylin and mounted on a cover slip. Evaluation of liver fibrosis was using Ishak fibrosis score.

### Cell culture

The rat HSC (HSC-T6) was purchased from ATCC and cultured in Dulbecco’s modified Eagle medium (DMEM) (Gibco, USA) supplemented with 10% fetal bovine serum (Gibco), 100 U/ml penicillin, and 100 mg/ml streptomycin. Primary rat HSCs were isolated from rat livers by pronase/collagenase digestion as previously described^10^. For each experiment, primary HSCs from 3 donor rats were used. HSC-T6 and primary HSCs were incubated with 5 ng/mL TGF-β1 in PBS buffer (R&D Systems, Inc., Minneapolis, USA) for 48 h for activation^11^.

### RNA extraction and quantitative real-time PCR (qRT-PCR)

miR-200a was isolated from cells or homogenized liver tissues using miRVana miRNA isolation kit (Takara, Dalian, China). Real time PCR assays were performed using PrimeScript^TM^ RT reagent Kits (TaKaRa), SYBR Green^®^ miRcute miRNA Realtime PCR Kit (Tiangen, Beijing, China), SYBR Green^®^ Realtime PCR Master Mix and Permix Ex Taq (TaKaRa) according to the manufacturer’s instructions.

Primers used were as follows: rat β-catenin, (Forward) 5′-CTT ACG GCA ATC AGG AAA GC-3′ and (Reverse) 5′-GAC AGA CAG CAC CTT CAG C-3′; GAPDH, (Forward) 5′-CGG ATT TGG TCG TAT TG-3′ and (Reverse) 5′-GAA GAT GGT GAT GGG ATT-3′. Primers for U6 and miR-200a were purchased from Sangon Biotech (Shanghai, China). The relative gene expression was normalized to the level of GAPDH while expression of miR-200a was normalized to the level of U6. All reactions were performed in triplicates for each sample. At least three independent experiments were carried out for each experimental condition.

### Enzyme linked immunosorbent assay (ELISA)

The cell culture supernatant was collected after 48 h culture. The concentrations of α-SMA and type I collagen (Col I) in the supernatant were measured using a ELISA kit (Cusabio, Wuhan, China) according to the manufacturer’s instructions. The optical density of the microplate was determined at 450 nm.

### Western blot analysis

After treatment with IL-22 for 48 h, the protein was extracted from cell lysates in the lysis buffer containing protease inhibitors and phosphatase inhibitors (Sigma, USA). The concentration of cellular protein was determined by the Pierce BCA assay (Thermo Fisher Scientific, Rockford, USA). Total protein extracts were separated by electrophoresis for 90 min and transferred onto a polyvinylidene fluoride membrane (Merck Millipore, USA). The primary antibodies used were rabbit anti-STAT3 (1:3000, Abcam, UK), anti-p-STAT3 primary antibody (1:2000, Abcam) and anti-β-catenin (1:2000, Abcam). LI-COR IRDye 680-labeled secondary antibody was used (Rockland Immunochemical, Gilbertsville, PA). The signals were detected and quantified by using Odyssey Infrared Imaging System (Li-COR Biosciences, Lincoln, NE) and Fluorchem 8900 system (Alpha Innotech, San Leandro, CA).

### Overexpression and knock down miR-200a in HSC

To overexpress and knock down miR-200a, lentiviruses of rno-miR-200a mimics, miR-200a inhibitors and a non-specific control were transduced into HSC cells (Genepharma, Shanghai, China). The sequences were as follows: miRNA-200a mimic sense 5′-UAA CAC UGU CUG GUA ACG AUG U-3′ and anti-sense 5′-ACA UCG UUA CC A GAC AGU GUU A-3′, and miRNA-200a inhibitor sequences were 5′-ACA UCG UUA CCA GAC AGU GUU A-3′. After transduction with rno-miR-200a lentivirus for 72 h, cells were monitored under immunofluorescence microscopy to ensure at least 80% of the cells expressed green fluorescence protein (GFP). qRT-PCR was used to detect the expression of miR-200a.

### Cell proliferation and apoptosis assay

The Cell Counting Kit-8 (CCK-8, Dojindo, Beijing, China) assay was used as a qualitative index of cell proliferation according to the manufacturer’s instructions. Apoptotic cells were quantified using FITC Annexin V Apoptosis Detection Kit (BD Biosciences, Vienna, Austria) according to the manufacturer’s instructions. Cell analyses were performed by BD FACSCalibur flow cytometer (BD Biosciences). The apoptotic cells were defined as Annexin- V-positive cells. Each experiment was repeated three times independently.

### Luciferase reporter assay

The sequences of β-catenin 3′-UTR containing the predicted miR-200a binding sites were cloned into psiCHECK2 (C8021, Promega, USA). The constructs were transfected into HEK293 cells together with miR-200a or miR-200a inhibitors by using Lipofectamine 2000 (Invitrogen). After transfection for 48 h, the Renilla and Firefly luciferase activities were measured by the Dual-Luciferase Reporter Assay (Promega, Madison, WI, USA) with a luminometer (SynergyTM 4 Hybrid Microplate Reader, BioTek, USA). The luciferase activity values were normalized using the Renilla values.

### Bioinformatic analyses

Three algorithms (TargetScan, Diana-microT and miRWalk) were used to predict miRNA targets. We restricted miRNA binding sites to the 3′UTR region. The minimum seed length for miRNA binding was 7 nucleotides.

### Statistical analysis

All data were analyzed using SPSS 16.0 software (SPSS Inc, Chicago, IL, USA). Data were expressed as means ± standard deviation (SD). Two group comparisons were carried out using Student’s t test or Mann–Whitney U test when appropriated. The differences among multiple groups were compared using one-way analysis of variance (ANOVA) followed by LSD post-hoc test. A two-sided *p* value of less than 0.05 indicated statistical significance.

## Results

### IL-22 suppressed HSC proliferation

**Figure 1:**
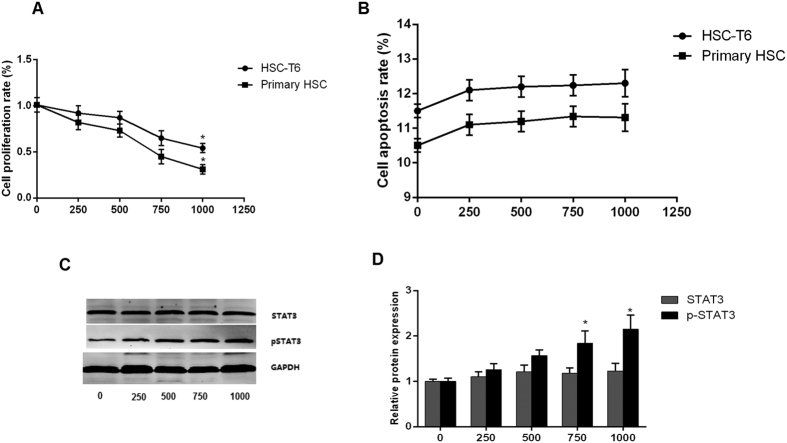
IL-22 inhibited HSC proliferation via STAT3 pathway. (**A**) IL-22 significantly inhibited proliferation of HSC; (**B**) No significantly change of apoptosis of HSC after IL-22 treatment. (**C,D**) Protein expression of STAT3 and phosphorylated STAT (p-STAT3) in HSC after treatments with different concentrations of IL-22. (n = 3). *p < 0.05.

HSC-T6 and primary HSC were incubated with recombinant IL-22 protein at concentrations of 250, 500, 750 and 1000 pg/mL respectively for 48 h. Treatment with higher concentration of IL-22 protein showed stronger inhibitory effect on the proliferation of HSC cell line and primary HSC (Fig. 1A). In contrast, the apoptosis rate of HSC did not change significantly in response to incubation with different concentrations of IL-22 protein (Fig. 1B). Treatment with either 750 or 1000 pg/mL IL-22 significantly induced expression of phosphorylated STAT (p-STAT3) but not STAT3 (Fig. 1C,D).

### Expression of miR-200a and β-catenin after HSC activation

**Figure 2:**
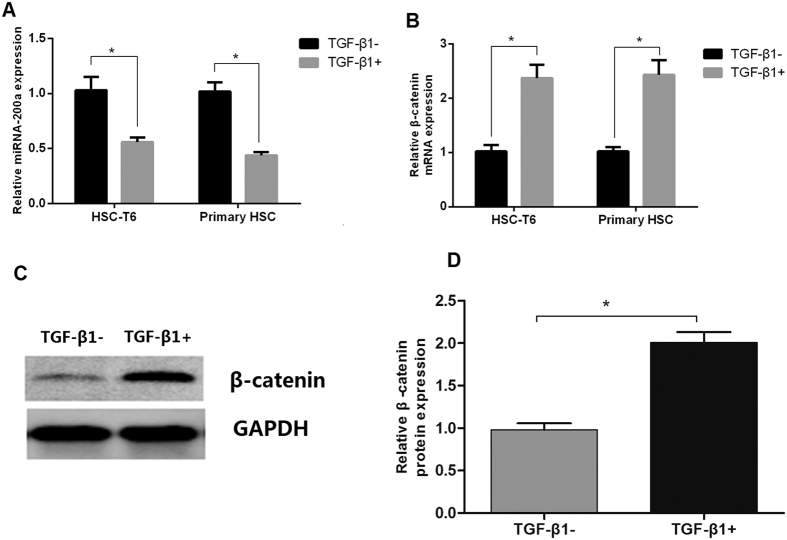
Expression of miR-200a and β-catenin was decreased during HSC activation. (**A**) Expression of miR-200a in HSC was decreased after treatment with TGF-β1; (**B**) Expression of β-catenin mRNA in HSC was increased after treatment with TGF-β1; (**C,D**) Western blotting indicated that β-catenin protein levels were increased in HSC after treatment with TGF-β1. (n = 3). *p < 0.05.

HSC-T6 and primary HSC were activated by incubation with 5 ng/mL TGF-β1 for 48 h. After HSC activation, expression of miR-200a remarkably decreased (Fig. 2A), while expression of β-catenin significantly increased in mRNA (Fig. 2B) and protein levels (Fig. 2C,D).

### miR-200a inhibited HSC proliferation

**Figure 3:**
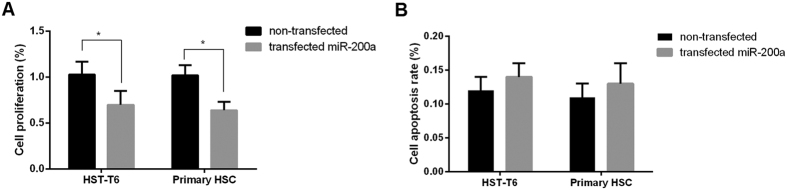
miR-200a inhibited proliferation of HSC. (**A**) Transfection with miR-200a mimics suppressed HSC proliferation; (**B**) No change of HSC apoptosis after transfection with miR-200a mimics. (n = 3). *p < 0.05.

Since HSC activation suppressed expression of miR-200a, we further overexpressed miR-200a with a lentiviral vector in HSC and measured its effect on proliferation and apoptosis. We found that overexpression of miR-200a inhibited not only HSC-T6 but also primary HSC proliferation (Fig. 3A). However, the overexpression had no effect on the apoptosis of HSC-T6 and primary HSC (Fig. 3B). Collectively, these results suggested an inhibitory role of miR-200a in HSC proliferation.

### β-catenin was a downstream target of miR-200a

**Figure 4:**
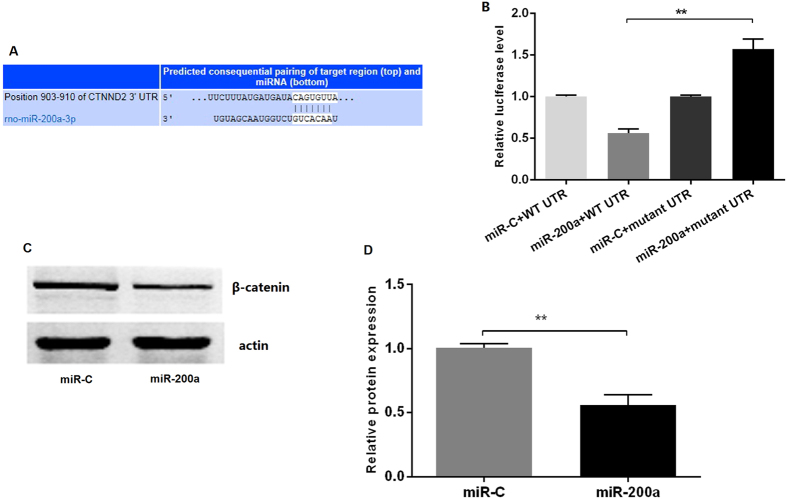
miR-200a targeting β-catenin in HSC. (**A**) The predicted miR-200a binding site at the 3′-UTR of β-catenin mRNA; (**B**) Luciferase reporter assay revealed that β-catenin is a target of miR-200a; (**C,D**) Western-blot indicated that miR-200a significantly decreased protein expression of β-catenin. (n = 3). **p < 0.01.

Target prediction for miR-200a suggested that it regulates the expression of β-catenin through a potential seed region in 3′UTR (Fig. 4A). To confirm that β-catenin is a direct target of miR-200a, we obtained luciferase-3′UTR reporter constructs for the mRNA and transfected them into HEK293T cells together with miR-200a mimics or a non-targeting control miRNA. Transfection with miR-200a significantly reduced firefly luciferase activity for β-catenin (*p* < 0.01, Fig. 4B) compared to the negative control. 3′UTR mutagenesis of sequence complementary to the miR-200a seed region attenuated miRNA effect (Fig. 4C,D), suggesting that β-catenin is a directly regulated by miR-200a in HSC.

### IL-22 inhibited HSC activity via regulation of miR-200a

**Figure 5:**
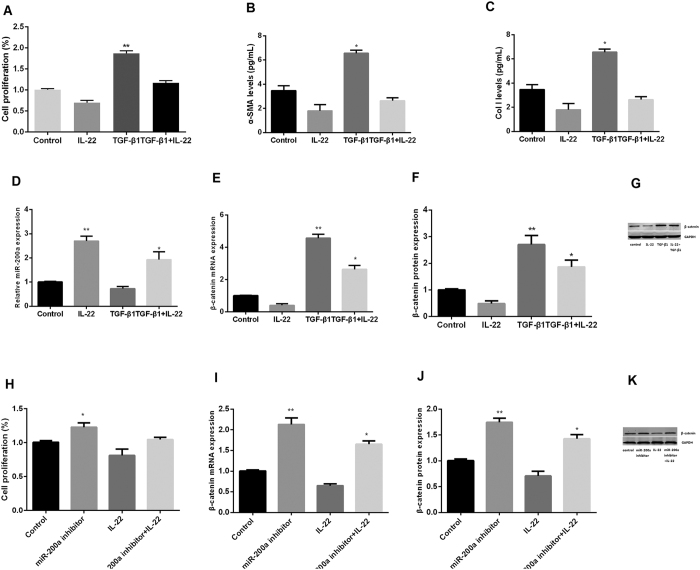
IL-22 inhibited HSC activation via regulation of miR-200a/β-catenin. (**A**) Proliferation of HSC after treatment with IL-22, TGF-β1 or the combination; (**B,C**) Expression of α-SMA and Col I in the supernatant; (**D**) Expression of miR-200a in HSC; (**E–G**): Expression of β-catenin mRNA and protein in HSC; (**H**) Proliferation of HSC after treatment with IL-22, miR-200a inhibitors or the combination; (**I–K**) Expression of β-catenin mRNA and protein in HSC after treatment with IL-22, miR-200a inhibitors or the combination. (one-way ANOVA/LSD post-hoc test, n = 3). *p < 0.05.

Primary HSC cells pre-incubated with TGF-β1 for 24 h and subsequently cultured with 1.5 ng/mL IL-22 for 48 h. For activated HSC cells, treatment with IL-22 significantly inhibited HSC proliferation (Fig. 5A) and suppressed mRNA levels of α-SMA and Col I in the supernatant (Fig. 5B,C), Of note, treatment with IL-22 induced mRNA expression of miR-200a (Fig. 5D) and decreased mRNA and protein levels of β-catenin in the activated HSC cells (Fig. 5E–G).

In order to explore the relationship between IL-22 and miR-200a in HSC activity, we knocked down miR-200a in HSC cells using its inhibitors, subsequently treated cells with IL-22 protein and measured cell proliferation, mRNA and protein expression. The proliferation of HSC was increased and then compared to that without miR-200a inhibitors transfection (Fig. 5H), and the expression of β-catenin increased significantly (Fig. 5I–K). Taken together, these results indicated that IL-22 inhibited HSC activity through the regulation of miR-200a/β-catenin.

### IL-22 alleviated liver fibrosis and increased miR-200a expression in rat

**Figure 6:**
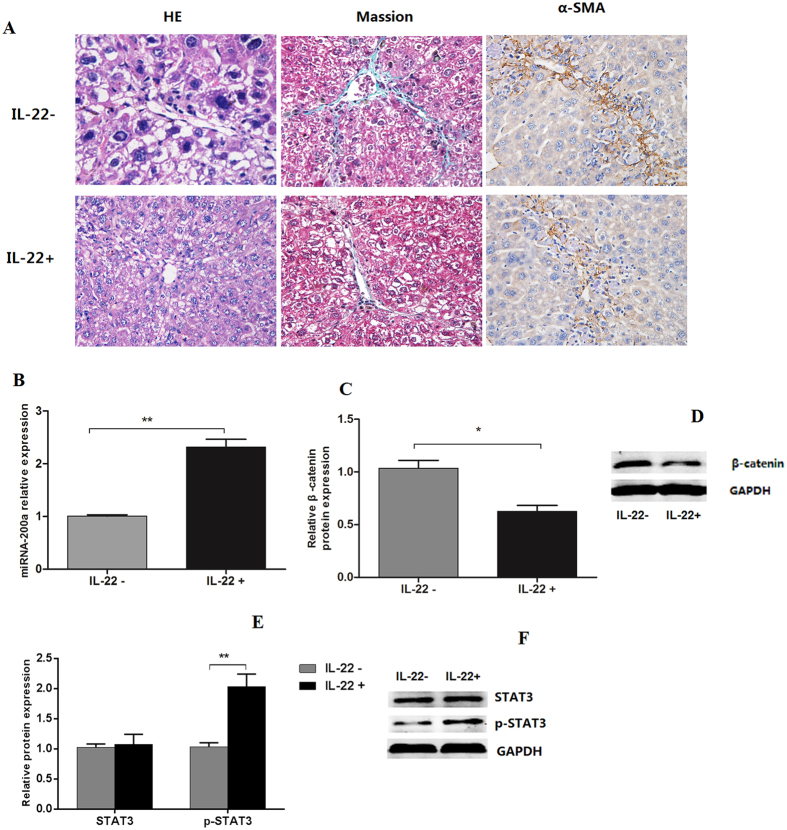
IL-22 alleviated liver fibrosis and increased miR-200a expression in rat. (**A**) Change of liver tissue of rat with liver fibrosis with or without IL-22 treatment. (**B**) Expression of miR-200a in liver tissue with or without IL-22 treatment; (**C,D**) Expression of β-catenin in liver tissue with or without IL-22 treatment; (**E,F**) Expression of STAT3 in liver tissue with or without IL-22 treatment. (n = 5). *p < 0.05, **p < 0.01.

To further elucidate the relationship between IL-22 and miR-200a in liver fibrosis, a fibrotic rat model was IP injected with rrIL-22. We observed that fibrotic status was alleviated after rrIL-22 treatment compared with controls (Fig. 6A). Moreover, IL-22 treatment induced expression levels of miR-200a (Fig. 6B) and reduced mRNA and protein expression of β-catenin in rat fibrotic liver compared with controls (Fig. 6C,D). Furthermore, we found expression levels of p-STAT3 significantly increased upon IL-22 treatment while STAT3 not (Fig. 6E,F).

### Effect of IL-22 on liver fibrosis was reduced by miR-200a inhibitor transfection

**Figure 7:**
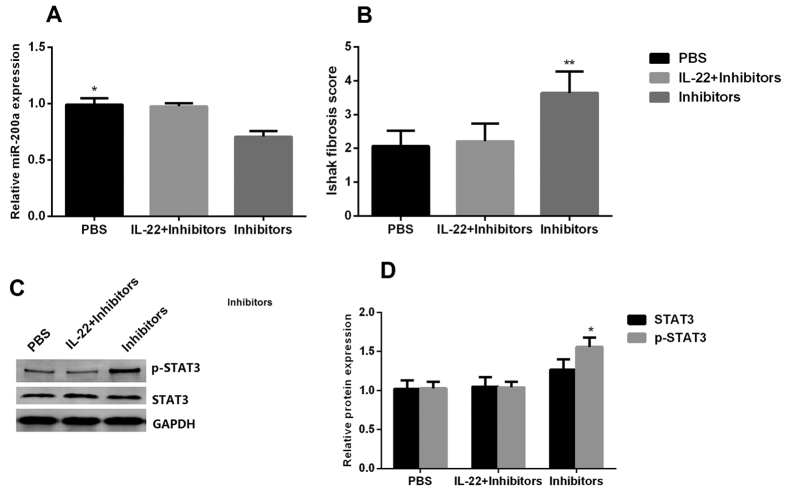
Effect of IL-22 on liver fibrosis was reduced by miR-200a inhibitor transfection. (**A**) Expression of miR-200a in rat fibrotic liver tissue in the PBS group, IL-22 + miR-200a inhibitors group and miR-200a inhibitors group; (**B**) Ishak fibrosis score in rat fibrotic liver tissue in the PBS group, IL-22 + miR-200a inhibitors group and miR-200a inhibitors group; (**C**,**D**) Expression of STAT3 and p-STAT3 proteins in fibrotic rat liver tissue in the PBS group, IL-22 + miR-200a inhibitors group and miR-200a inhibitors group. (one-way ANOVA/LSD post-hoc test, n = 5). *p < 0.05, **p < 0.01.

The expression levels of miR-200a in rat fibrotic liver significantly decreased in the group treated with miR-200a inhibitor injection compared with PBS group and IL-22 + miR-200a inhibtors group (Fig. 7A). Similar to the results of expression of miR-200a, we found that the liver fibrosis recovery slower after miR-200a inhibitor injection compared with PBS group and the IL-22 + miR-200a inhibitor group according to the Ishak fibrosis score (Fig. 7B). We also observed that the expression of p-STAT3 was increased in the group of miR-200a inhibitor injection compared with PBS group and IL-22 + miR-200a inhibitors group (Fig. 7C,D). Collectively, these results suggested that miR-200a was involved in the regulatory function of IL-22 in alleviating liver fibrosis.

### Interaction of β-catenin and STAT3 in HSC

**Figure 8:**
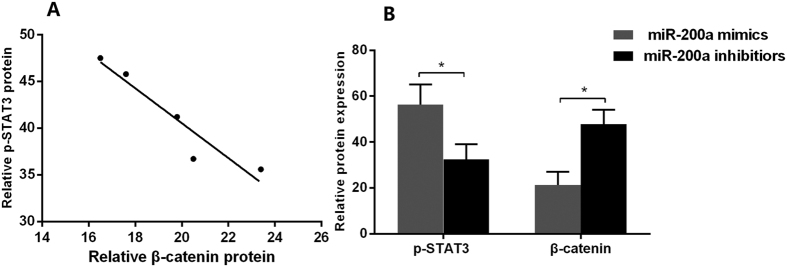
β-catenin and STAT3 protein expression in response to miR-200a in HSC. (**A**) Correlation analysis of p-STAT3 and β-catenin after treatment with IL-22 in HSC; (**B**) Comparisons of STAT3 and β-catenin protein levels in HSC after miR-200a mimics or miR-200a inhibitors transfection. (n = 3). *p < 0.05, **p < 0.01.

Expression of p-STAT3 significantly increased while β-catenin decreased along with the treatment with IL-22 concentration from low to high concentrations in HSC. The protein concentrations of p-STAT3 was inversely correlated that of β-catenin (*P* = 0.014, Fig. 8A).

Using miR-200a mimics to transfect HSC, we found that the expression of p-STAT3 was increased in HSC with the overexpression of miR-200a, which was contrast to the expression of β-catenin, and transfection of miR-200a inhibitors in HSC resulted in decreased of p-STAT3 and increased of β-catenin (Fig. 8B).

## Discussion

IL-22, a member of IL-10 cytokine family, is produced by Th17, Th22 cells^12^ and activated NK and NKT cells^13^. IL-22 exerts its cellular effects via a heterodimeric transmembrane receptor formed by IL-10R2 and IL-22R1^14^, subsequently activates Janus kinase-signal transducers and activators of transcription molecules including STAT3, Jak1 and Tyk2^15^. To date, several studies reported potential effects of IL-22 on liver fibrosis; however, the mechanism of IL-22 in liver fibrogenesis remains unclear^16^,^17^.

In agreement with previous reports^3^,^4^, our data showed that treatment with IL-22 significantly inhibited development of liver fibrosis. Moreover, similar to the Kong *et al.*^3^ report, we found that inhibitory effect of IL-22 on HSC was majorly reflected by suppression of cell proliferation rather than apoptosis, and reduction of cytokines, α-SMA and Col. We further observed that STAT3 pathway was activated along with the treatment, indicating that IL-22 may inhibit HSC proliferation through STAT3 pathway. It was reported that STAT3 activation in response to IL-22 induces HSC senescence and subsequently inhibits HSC proliferation^3^. Moreover, IL-22/STAT3 has an anti-fibrotic function in T cell-mediated murine hepatitis. Radaeva *et al.*^18^ showed that IL-22 blockade reduced STAT3 activation and exacerbated liver injury in T cell-mediated hepatitis, whereas injection of IL-22 attenuates the injury. Blocking STAT3 activation abolishes the antiapoptotic and mitogenic actions of IL-22 in hepatic cells.

To date, a few miRNAs have profound role in the development of liver fibrosis. The miRNA-200 family was reported to be an important one in liver fibrogenesis^19^,^20^. A recent study showed that miRNA-200a silencing activated HSC through Keap1, while overexpression of miRNA-200a inhibited HSC proliferation^8^. Consistent with Yang *et al.*^8^ study, we found that expression of miRNA-200a was reduced in the activated HSC as well as in fibrotic rat liver tissues compared with controls, suggesting that loss of miRNA-200a induces development of liver fibrosis.

β-catenin, a component of adhering junctions, is necessary for cadherin–catenin interaction^21^. As a key signaling effector in the Wnt signaling pathway, β-catenin is involved in the cell survival, proliferation, migration and polarity^22^. Several studies reported that β-catenin was overexpressed in human tissues with liver fibrosis while blockage of Wnt/β-catenin pathway inhibited HSC activation^23^,^24^,^25^,^26^. Our study identified β-catenin as a direct target of miRNA-200a, suggesting that miRNA-200a exerts its anti-fibrotic effects via directly down-regulated β-catenin expression.

Liver fibrogenesis consists a number of critical pathways that may interact together as functional partners. For example, interaction between Notch Hedgehog pathways controls the cell fate and mediates liver repair *in vivo*^27^. Cross-talk between TGF-β1 and epidermal growth factor receptor signaling pathways induces TM4SF5 expression and epithelial-mesenchymal transition^28^. Our study showed treatment with IL-22 induced the expression of miR-200a, decreased β-catenin in HSC and rat fibrotic liver tissues via STAT3 pathway. We further identified the inverse association between expression of STAT3 and β-catenin in HSC, indicating there may have a cross-talk between IL-22/STAT3 and Wnt/β-catenin signaling pathways in development of liver fibrosis. Further study is warranted in understanding how these two pathways are connected.

In conclusion, our results demonstrate that IL-22/STAT3 regulates HSC activation and ameliorates liver fibrosis through modulating expression of miR-200a and β-catenin. The interaction between IL-22/STAT3 and Wnt/β-catenin pathway is involved in the development of hepatic fibrosis *in vivo*. These results might help in developing more effective therapy for liver fibrosis.

## Additional Information

**How to cite this article**: Hu, B. *et al.* Interleukin-22 ameliorates liver fibrosis through miR-200a/beta-catenin. *Sci. Rep.* **6**, 36436; doi: 10.1038/srep36436 (2016).

**Publisher’s note:** Springer Nature remains neutral with regard to jurisdictional claims in published maps and institutional affiliations.
